# Learning Entropy as a Learning-Based Information Concept

**DOI:** 10.3390/e21020166

**Published:** 2019-02-11

**Authors:** Ivo Bukovsky, Witold Kinsner, Noriyasu Homma

**Affiliations:** 1 Department of Mechanics, Biomechanics, and Mechatronics, Research Centre for Low-Carbon Energy Technologies, Faculty of Mechanical Engineering, Czech Technical University in Prague, Technicka 4, 166 07 Prague 6, Czech Republic; 2 Department of Electrical and Computer Engineering, University of Manitoba, Winnipeg, MB R3T 5V6, Canada; 3 Department of Radiological Imaging and Informatics, Tohoku University Graduate School of Medicine, Intelligent Biomedical System Engineering Laboratory, Graduate School of Biomedical Engineering, Tohoku University, Sendai 980-8575, Japan

**Keywords:** learning, information, novelty detection, non-probabilistic entropy, learning systems

## Abstract

Recently, a novel concept of a non-probabilistic novelty detection measure, based on a multi-scale quantification of unusually large learning efforts of machine learning systems, was introduced as learning entropy (LE). The key finding with LE is that the learning effort of learning systems is quantifiable as a novelty measure for each individually observed data point of otherwise complex dynamic systems, while the model accuracy is not a necessary requirement for novelty detection. This brief paper extends the explanation of LE from the point of an informatics approach towards a cognitive (learning-based) information measure emphasizing the distinction from Shannon’s concept of probabilistic information. Fundamental derivations of learning entropy and of its practical estimations are recalled and further extended. The potentials, limitations, and, thus, the current challenges of LE are discussed.

## 1. Introduction

Complexity measures and novelty detection measures, which are based on Shannon’s entropy [1], are probabilistic measures that do not consider the governing laws of systems explicitly. On the contrary, computational learning systems can approximate at least the contemporary governing laws of dynamical behavior. Novelty detection in dynamical systems is approached either by probabilistic approaches (e.g., [2]) or by utilization of learning systems, e.g., [3].

As the representative examples of probability-based novelty detection approaches, i.e., the statistical novelty measures and probabilistic entropy measures, we should mention sample entropy (SampEn) and approximate entropy (ApEn) [4,5]; SampEn and ApEn relate to fractal measures and thus to multi-scale evaluation [6,7,8,9] that is based on the concept of power-law [10]. The benefits of these multi-scale techniques were also shown via works on coarse-graining extensions to SampEn in [11,12] and recently also in [13]. Further, compensated transfer entropy [14] is another probabilistic technique for entropy evaluation via the conditional mutual information between present and past states. The probabilistic entropy approach for fault detection was published in [15] and probabilistic technique for sensor data concept drift (also concept shift) appeared in [16].

Among the probabilistic novelty approaches, we shall also mention the currently popular concepts of generalized entropies, especially, the extensively studied Tsallis and Rényi entropies and their potentials, e.g., [17] and references therein. The example of their application, e.g., to probabilistic anomaly detection in cybersecurity, can be found in [18]. In learning systems such as neural networks, the generalized entropies are also naturally studied to improve the learning process, e.g., [19]. As regards the proposed learning entropy (LE), it could also be used in the difficult problem of anomaly detection during a cyberattack, e.g., a system has been developed to detect such anomalies using convolutional neural networks and multi-scale and poly-scale measures [20]; adding LE to such a system could enhance the real-time detection of DDOS and other attacks. However, LE is a non-probabilistic measure that evaluates unusually large learning efforts of a learning system, so it is also different from the studied applications of generalized entropies.

The second direction of novelty detection in dynamical systems, i.e., the direction of non-probabilistic novelty measures, is based on learning systems that employ machine learning, and we apply this direction in our research too. As some more recent survey works on non-probabilistic detection methods with learning systems, we may refer to [21,22,23,24] and to [25] as to an example that involves incremental learning. The usage of residuals of learning system output for fault detection with nonlinear estimators was studied in [22,23,24]. For recent works on adaptive concept drift detection, we may refer to works [25,26,27,28] and to newer works in area of neural networks [29,30,31] and [32] as a work on a cognitive system for sensor fault diagnosis. A certain similarity with our proposed concept can also be found in the adaptive resonance theory [33].

While the probabilistic approaches do not explicitly reflect the governing law of data, the learning-system-based methods rely on the evaluation of model residuals and thus on certain accuracy of models. However, there has been a missing concept for novelty detection that would utilize the learning process without probability computations and that would not rely on the accuracy of learning systems, and learning entropy is such a novel concept in this sense.

The motivation of this brief paper is to discuss and extend the recently introduced concept of LE [34,35] in the sense of a (machine) learning-based information measure as a founding concept of the cognitive novelty detection based on quantification of unusual learning efforts of learning systems. Novelty detection via LE is based on real-time learning of systems after they had been pretrained on an initial pretraining data set. Aside from the founding work [34], other examples of works that indicate the usefulness of LE in biomedical or technical data and for novelty detection in data with concept drift can be found in [36,37,38,39].

Section 2 first discusses a loose parallel between entropy in the sense of thermodynamics and importantly, the distinction from the concept of Shannon’s information theory, with the proposal of the concept of (machine) learning-based information measure. Secondly, the original multiscale LE algorithm [34,35] based on unusually large learning efforts is reviewed and followed by its approximate version. Thirdly, it provides an alternative (more direct) formula for practical computations.

In the following text, terms such as learning system, neural network, model, observer, and predictor are used interchangeably, unless it is stated otherwise. To simplify mathematical notations, the discrete time index *k* is dropped from notations unless it is necessary for clarity.

## 2. Concept of Learning Information Measure

A loose parallel between entropy concepts of informatics and thermodynamics can be drawn regarding learning systems and training data. More novel data carries more information from the point of view of a learning system. When novel training data are presented to the learning system (after its pretraining), then the learning algorithm responds via its learning activity with its adaptive parameters. Hence, the information (novelty) that training data means to the learning system changes the activity of learning system, similar to how heat changes the energy of thermodynamical systems. For incrementally learning systems with a vector of all adaptive parameters w, the novelty in data can change the actual learning effort, so the actual weight updates Δw or at least some of them, indicate the novelty that the data provides to the contemporary trained learning systems. The weight updates represent additional information for a better description of real systems by neural networks (or learning systems in general). These loose analogies between the novelty of training data, learning effort, and weight updates are the necessary information elements to improve system description, as well as to draw connotations from the concept and meaning of entropy in a general sense, including those of thermodynamics and information theory (a review on meanings of entropy can be found in [40]).

According to Shannon’s probabilistic approach, the amount of self-information I that the value y(k) can provide to an observer, depends on its inverse probability as follows:

(1) I(y(k))=−log(p(y(k))).

where p(y(k)) is the probability of value y(k) that is in fact independent of the discrete time index k, and the less frequent value of y(k) the larger information it provides to an observer. However, if the observer is a learning system that learns the governing law of data, then the statistically new data do not necessarily provide new information (i.e., as with the non-repeating, yet deterministic chaos). The statistically new data can still comply with the temporarily learned governing law, so the learning system is not “surprised” by its appearance. This points us to the essence of calculating the novelty (information) that data provide to an observer in a different way than that established via the Shannon probabilistic sense as in (1). While the probabilistic information measure is based on clustering that utilizes a distance between vectors of data, we may quantify the familiarity of a learning-system with data because the learning system considers data to be novel if the data do not comply with the contemporary learned governing law via the following:

- Supervised learning (as for given input–output patterns with supervised learning), or via
- unsupervised learning (such as learned by clustering methods, SOMs, or auto-encoders).

**Figure 1:**
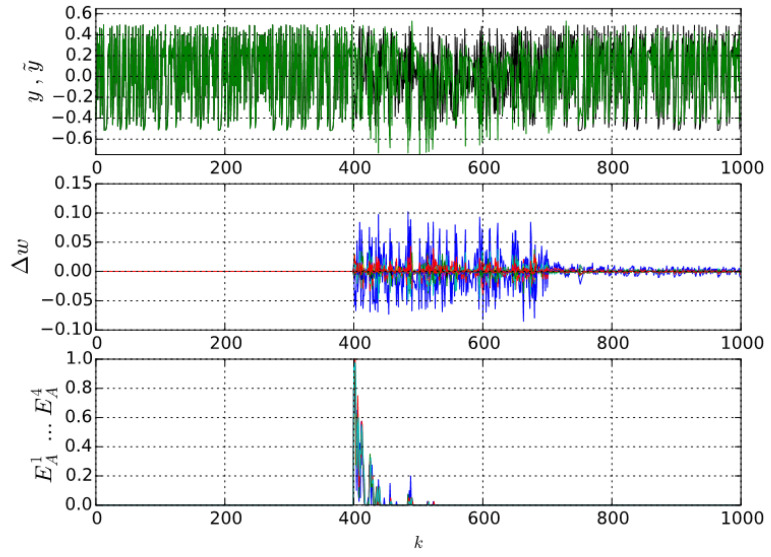
(**Top**) Chaotic (deterministic) time series with a sudden occurrence of white noise (k > 400) superimposed on the output of its real-time sample-by-sample learning predictor. (**Middle**) The weight updates cannot converge to noise. (**Bottom**) Approximate Learning Entropies (of various orders) via (19) detect the noise as the novelty immediately at its occurrence at k > 400 and then LE decreases as the large variance of learning increments becomes a new usual learning pattern (details on LE and its orders can be found in Section 4.1 and Section 4.2).

The most straightforward way to quantify novelty with supervised learning is to use a model (e.g., prediction) error that indicates the expectancy of the actual data from the governing-law viewpoint. However, this assumes to have a correctly designed learning model that is not trivial to obtain for the real-world data. In fact, the (prediction) error is not the most straightforward quantity that either tells us how much information the learning system needs, or how much learning effort it is going to spend to become more familiar with the new data. The higher error does not necessarily mean that the actually presented data are novel because the model can be limited in the quality of approximation, and its generalization capability is unknown for data that never occurred before. Also, the model error is only one component of the learning algorithm and each model parameter can be updated with different significance and magnitude, depending on other factors including inputs. During sample-by-sample or sliding-window batch pretraining on the initial training dataset, the weights become updated with smaller and smaller updates in each consecutive training epoch, so the parameters of a learning system converge up to a certain pattern of behavior. Thus, for the pretraining dataset, the average update magnitudes of individual weights finally become constant, i.e., $\bar{\left|\Delta w\right|}=\mathrm{const}.$. If retraining continued for further data that comply with the pretrained governing law, then in principle, further weight updates of a pretrained learning system would not be larger than those during pretraining (even if the model could not learn the governing law properly). However, if the retraining data involve data samples that do not comply with the temporarily learned governing law, the weight update behavior changes as the learning system tends to get adapted to novel data and weight updates can be larger (see middle axes in Figure 1 for k≥400).

Thus, the learning updates Δw represent learning effort and they are suitable for evaluation of how much information the new data convey to a learning system in terms of the contemporary learned governing law. In particular, if all weights are updated within the usually large magnitudes, then the retraining data do not bring any new information to the learning system. However, if more weights are updated with unusual updates, the data appear to be more unexpected, thus leading to a more unusual learning effort. This also means that data convey more information to the learning system. Thus, the detection of unusual weight updates can be used to detect novel data, and naturally the higher count of unusual updates the more information the retraining data convey to the already pretrained model. Then, a (machine) learning-based information measure can be generally proposed via a suitable aggregation of unusual learning increments as follows:

(2) L(k)=A(f(Δw(k)))

where A(.) represents a general aggregation function and f(.) denotes a function that quantifies the unusuality of the learning effort via learning increments (assuming the learning system has been pretrained on the training data). So far in our research of LE [34,36,38,41,42,43], a summation has been applied as the aggregation function A(.) as follows:

(3) $$L(k)=\sum_{\forall \Delta w\in \Delta w}f(\Delta w(k))\;[/]$$

and f(.) for detection of unusually large learning effort has been defined via unusually large weight increments as follows

(4) $$f(\Delta w)=\{\begin{array}{ll} 1 & \mathrm{if}\;\left|\Delta w\right|\;\mathrm{is}\;\mathrm{unusually}\;\mathrm{large}\;\\ 0 & \mathrm{if}\;\left|\Delta w\right|\;\mathrm{else} \end{array}$$

In reality, it is practically impossible to choose the best bias that determines the unusually large weight update magnitudes for proper evaluation of (4), so the detection sensitivity for unusually large weight updates was resolved via a power-law based multi-scale approach as in [34,43] and that is reviewed and modified in later sections.

## 3. Shannon Entropy versus Learning Entropy

Until now, we have discussed the Shannon entropy, i.e., the probabilistic, information measure I (1) vs. the learning-system-based concept of information measure L (3) and (4). Both I(y(k)) and L(y(k)) represents the quantity of how unusual data sample y(k) is. However, we cannot think about L in the sense of histogram-bin clustered data, because while for the Shannon concept I it holds that

(5) $$y(k_{1})=y(k_{2})\Rightarrow I(y(k_{1}))=I(y(k_{2}))=I_{i}$$

where *i* denotes the bin index, the learning measure L is likely to be different for two identical values of data at different times because of the learning process; i.e.,

(6) $$y(k_{1})=y(k_{2})⇏(y(k_{1}))=L(y(k_{2}))\;$$

Thus, it is apparent from (5) and (6) that the Shannon entropy definition, i.e., the probability-weighted average of the information measure

(7) $$H=\sum_{i}p_{i}\cdot I_{i}$$

where i denotes the normalized histogram bin index, and cannot be used in the same way for the learning-based measure L. In light of the learning-based information measure L and its distinction from the Shannon measure I, a multiscale extension of L via (3) and (4) was introduced as the approximate individual sample learning entropy (AISLE) in [34] (for more details, see Section 4 below). AISLE reflects the amount of the unusually large learning effort that learning system spends on updating to novel data, and thus it reflects the amount of new information that data means to a learning system (or loosely such as the energy with which novel data boosts the learning engine).

The most straightforward measure based on AISLE is the learning entropy profile (LEP) that was defined in [34] as the cumulative sum of LE in time over the whole interval of data as follows

(8) $$LEP=\sum_{k=1}^{N}LE(k)\approx \sum_{k=1}^{N}L(k)$$

Thus, the LEP is a function that quantifies the novelty that a pretrained learning system is able to find in a new dataset in terms of its unusual learning effort. The last point of LEP is called the learning entropy of a model (LEM)

(9) LEM=LEP(k=N)

In other words, LE characterizes how pretrained neural network is unfamiliar with each new data point (in time), while the LEP quantifies the total amount of novelty that the interval of data has conveyed to the pretrained learning system, and it also gives a notion about the novelty (learning information) in data from the point of learning effort for the used mathematical structure and its particularly used learning algorithm. Based on incremental learning (11), we can see from (3), (4), (8) and (9) that the learning entropy of a model is always increasing.

## 4. Algorithms for Learning Entropy Estimation

The previous sections recalled the concept of LE and discussed it with connotation to a (machine) learning-based information concept. Further, the theoretical multiscale algorithm for the estimation of LE is reviewed in Section 4.1, followed with practical formula in Section 4.2 with new direct formula in Section 4.3.

### 4.1. The Multiscale Approach

A general form of a learning system (LS) is as follows:

(10) $$\tilde{y}=F(w,u)$$

where $\tilde{y}$ is the vector of actual outputs, **u** is the vector of inputs (including feedbacks in case of a recurrent learning system), F(.) is the general mapping function of LS, and **w** represents the vector of all adaptable parameters (weights). Further derivations apply when the learning entropy considers all neural weights in **w**; however, customization of the algorithm for individual weights may be an interesting research challenge, particularly for deep neural networks. Further for simplicity, let us assume that all neural weights are updated at the same time according to the incremental scheme as follows

(11) w(k+1)=w(k)+Δw(k)

where Δw(k) is the vector of actual weight updates that depend on a particularly chosen learning algorithm and its potential modification. The concept of learning entropy is based on the evaluation of unusual weight updates as the unusual learning pattern can indicate novelty in training data; i.e., the new information that new samples of data carry in respect to what the NN contemporary has learned already [34]. This methodology to evaluate the learning entropy through the unusually large weight updates was recently introduced [34] and then reviewed with some simplifications recently in [35,36,38]. The first important parameters here are as follows:

- α is the relative detection sensitivity parameter that defines the crisp border between usual weight updates and unusually large ones (since the optimal *α* is never known in advance, the multi-scale evaluation has to be adopted).
- M is the length of the floating window over which the average magnitudes of the recent weight updates are calculated (for periodical data, there is also the lag *m* between the actual time and the end of the window, see p. 4179 in [34]),

Then the unusual learning effort of LS can be evaluated at each learning update (through (11)) as the count of unusually large weight increments for all weights of the LS as follows:

(12) $$L(\alpha )=\sum_{\forall \Delta w\in \Delta w}f(\Delta w(k),\alpha )$$

where f(.) is the detection function defined for every individual weight increment as follows:

(13) $$f(\Delta w,\alpha )=\left\{ \begin{array}{l} 1\;\mathrm{if}\;\left(\left|\left|\Delta w\right|-\bar{\left|\Delta w\right|}\right|\right)>\alpha \cdot \sigma_{\Delta w}\\ 0\;\mathrm{else} \end{array}\right.$$

where the detection sensitivity α is defined above, $\sigma_{\Delta w}$ is the standard deviation of recently usual weight update magnitude, and the average weight-update magnitude can be calculated as follows:

(14) $$\bar{\left|\Delta w\right|}=\bar{\left|\Delta w^{M}\right|}=\frac{1}{M}\sum_{j=k-M-m}^{k-1-m}\left|\Delta w(j)\right|$$

where M is the length of the floating window and m is the optional lag for data with features of periodicity (as indicated in Equation (27) in [34]. Notice, we should calculate $\bar{\left|\Delta w\right|}$ when a learning system had been already pretrained in such a way so learning does not display any more convergence (LE is attractive also for that it is principally independent of any model accuracy [43], while the pretraining and further learning are the key principles of LE).

Since the count of all unusual weight updates L(k,α) depends on detection sensitivity α, and since we do not know the optimal sensitivity for the particular learning system (i.e., for the particular LS, or the learning algorithm used, or for the data) we shall overcome this single scale issue by using a multi-scale approach that evaluates the unusual learning effort over the whole interval of detection sensitivities α∈**α**. Considering that the real-word quantities non-linearly depend on parameters and being inspired by the use of the power-law from fractal analysis, we can assume that the dependence of the count of unusual weight updates on the detection sensitivity can be characterized via exponent *H* in the power-law relationship as follows:

(15) $$L(\alpha )≅{\left(\alpha \right)}^{-H}\;\Rightarrow \;log\left(L(\alpha )\right)≅-H\cdot log(\alpha )$$

and the characterizing exponent *H* then can be estimated as the slope of the log-log plot as

(16) $$H=\underset{\alpha \to \alpha_{max}^{-}}{\lim}\left(-\frac{log\left(L(\alpha )\right)}{log(\alpha )}\right)$$

where $\alpha_{max}$ was defined in [34] as the value where first unusual weight updates can be detected within all data. Alternatively, $\alpha_{max}$ is defined as follows:

(17) $$\alpha_{max}:\left\{ \begin{array}{l} \;\mathrm{if}\;\alpha \;>\alpha_{max}\Rightarrow \;\sum_{\forall k}L(\alpha ,k)=0\\ \;\mathrm{else}\;\sum_{\forall k}L(\alpha ,k)\;\geq \;1\; \end{array}\right.$$

Finally, we arrive at the definition of the learning entropy *E* as the normalized measure of unusually large learning effort at every weight update as follows

(18) $$E(k)=\frac{2}{\pi }\cdot \arctan\left(H(k)\right)\Rightarrow E(k)\in \left[0,1\right)$$

where E=0 means that no learning updates of all parameters are unusually large ∀α∈**α** and E→1 as all learning updates of all parameters are unusually large ∀α∈**α**. In fact, the learning entropy *E* in (18) is considered to be the first-order learning entropy because the detection function (13) is calculated with the first difference (≈ first-order derivative) of weights (as it results from (11)). It appeared useful to practically enhance LE computation with higher-order differences of weight updates that contribute to more reliable novelty detection as the higher order weight difference terms indicates useful noise filtering [34,35,36,38]. To compute the LE of various orders, the corresponding weight differences can be used in formulas (12)–(14) as in Table 1.

It should be emphasized, that the first important factor that affects the quality of the use of LE for novelty detection (i.e., for detecting data samples or intervals that carry new information that the neural network is not yet familiar with), is the proper pretraining of the neural network (e.g., an initial data set for further use online). In this case, the proper pretraining can be defined as such a long or repeated training as long as a learning performance index decreases, i.e., until the learning system tends to learn from data. In general, of course, the quality of adaptive novelty detection using the above derived LE further depends on the particularly chosen type of learning system, on the selected learning rule, on other setups that can be optimized the better we understand LS, the process, and its data.

This section recalled the theoretical derivation of learning entropy based on the fractal characterization of the power-law relationship of increased learning effort with a multiscale setup of detection parameter sensitivity α. The next section recalls a practical algorithm for the estimation of LE via cumulative sums and then a new direct algorithm based on the z-scoring of the temporal matrix of learning increments is introduced.

### 4.2. Practical Algorithm for Learning Entropy

The theoretical derivation of learning entropy (18) in Section 4.1 is based on estimating the characterizing exponent *H* as the slope of the log-log plot. In works [34,43], the calculation of characterizing exponent *H* of a log–log plot was replaced by the sum of quantities L(α) calculated for multiple values of detection sensitivities α and for all neural weight, so the learning entropy can be approximated as follows

(19) $$\begin{array}{c} E\approx E_{A}=\frac{1}{n_{\alpha }\cdot n_{w}}\;\sum \left\{L(\alpha );\;\alpha \in \alpha \right\}\\ \alpha \in \alpha =[\alpha_{1}<\alpha_{2}<\ldots <\alpha_{n_{\alpha }}],E_{A}\in \langle 0,1\rangle \end{array}$$

where E=0 means that no learning updates of all parameters are unusually large for any sensitivity α, and E=1 means that all learning updates of all parameters are unusually large for all sensitivities α, and where the sum is normalized for the length of vector **α** and for the total number of neural weights $n_{w}$, and thus (19) represents an approximation of LE. Particularly in [34], it is shown that the sum of L(α) along given by formula (12) in principle correlates to the log–log plot slope *H* calculated by formula (16). In particular, the steeper slope *H* is in a log–log plot, the more the L(α) counts increase along sensitivities α∈α, and that naturally results in the largest sum for most novel samples in data because L(α) starts increasing as soon as the neural network is learning more novel data. It is not necessary to find the exact value of $\alpha_{max}$ (see (16) and (17)), because α can in principle contain even larger values of α when calculated by (19). Thus, $E_{A}$ in (19) was introduced as approximate individual sample learning entropy (AISLE) when the sample-by-sample adaptation learning rule is used; e.g., the gradient descent learning in [34] and it was used also in works [35,36,38]. An example of AISLE of various orders is shown in Figure 1.

### 4.3. A Direct Algorithm

With mathematical symbols for the mean such as $\bar{x}$, for standard deviation as σ(x), and considering (14) introduces a special Z-scoring as follows:

(20) $$z(|\Delta w_{i}(k)|)=\frac{|\Delta w_{i}(k)|-\bar{\left|\Delta w_{i}^{M}(k-1)\right|}}{\sigma (|\Delta w_{i}^{M}(k-1)|)}$$

then a new formula for the estimation of LE can be introduced as an alternative to AISLE from (19) as follows:

(21) $$E(k)=\sum_{i=1}^{n_{w}}z(|\Delta w_{i}(k)|)\;;\;E\in \mathbb{R}$$

In contrast to previously proposed formulas for LE (18) and (19) that involved only the occurrences of unusually large learning efforts, the new direct formula (21) has the potential to quantify both unusually large learning efforts as well as unusually small ones; i.e., when the novelty in data makes weights become rapidly converging so their updates yield is unusually small in time and thus (21) results in unusually small values (see Figure 3 and the discussion there). Nevertheless, the novelty in data may be potentially detected even when only very few weight updates (or even a single one) unusually largely increase, and this makes LE be a very sensitive method. However, in principle, this is not well detectable by the LE formula (21) because the other weight updates may result in a negative contribution to E as $z(|\Delta w_{i}(k)|)<0$ for some i. Since (21) can also result in negative values of E (when the majority of weights are usually updated, or with even smaller updates), it would not provide a sharp border between usual and unusual learning effort. Thus, we can enhance (21) as follows

(22) $$\begin{array}{c} E(k)=\sum_{\forall w}\max\{0,z(|\Delta w(k)|)-\beta \};\;\\ E(k)\in \langle 0,+\infty ) \end{array}$$

that both

- detects only unusually large weight-update increments, larger than their recent mean plus β× standard deviation, and
- also directly computes their absolute significance (due to Z-scoring) for each weight while it was calculated in the previous concept of LE (18) and (19) via the multiscale evaluation over sensitivity setups (as recalled in Section 4.1 and (19)).

In order to achieve a normalized value of *E* in (22) as well as to cope with the single-scale issue of selection β, we propose to estimate the *r*-th order LE with this direct approach using a threshold function f(.) as well as multiple setups of sensitivity β as follows

(23) $$\begin{array}{c} E_{A}(k)=\frac{1}{n_{\beta }\cdot n_{w}}\sum_{\forall w}\sum_{\forall \beta }f\left(\max\{0,z(|\Delta w(k)|)-\beta \}\right);\\ \beta \in \beta =\left[\beta_{1}\;\beta_{2}\;\ldots \;\beta_{n_{\beta }}\right],\;f(.)=\{\begin{array}{l} 1\;\mathrm{if}\;(.)=True\\ 0\;\mathrm{if}\;(.)=False \end{array}\\ E(k)\in \langle 0,1\rangle \end{array}$$

where again $E_{A}=0$ means that no learning updates of all parameters are unusually large ∀β∈β, and $E_{A}=1$ means that all learning updates of all parameters are unusually large ∀β∈β. Furthermore, β represents a parameter of detection sensitivity that is related to the standard deviation of recent weight-update magnitudes and it causes formulas (22) and (23) work as follows:

- if β=0, the weight-update magnitudes larger than their recent mean are summed in (22) or counted in (23), i.e., the detection of unusual learning effort is the most sensitive one,
- if β=1, only the weight-update magnitudes larger than their recent mean plus one standard deviation are summed in (22) or counted in (23), i.e., the detection of unusual learning effort is less sensitive,
- if β=2, only the weight-update magnitudes larger than their recent mean plus two standard deviations are summed in (22) or counted in (23), i.e., the detection of unusual learning effort is even less sensitive, and, similarly, the detection of unusually large learning effort is less sensitive with the increasing parameter β while the vector of detection sensitivities β must not necessarily be a vector of integers.

**Figure 2:**
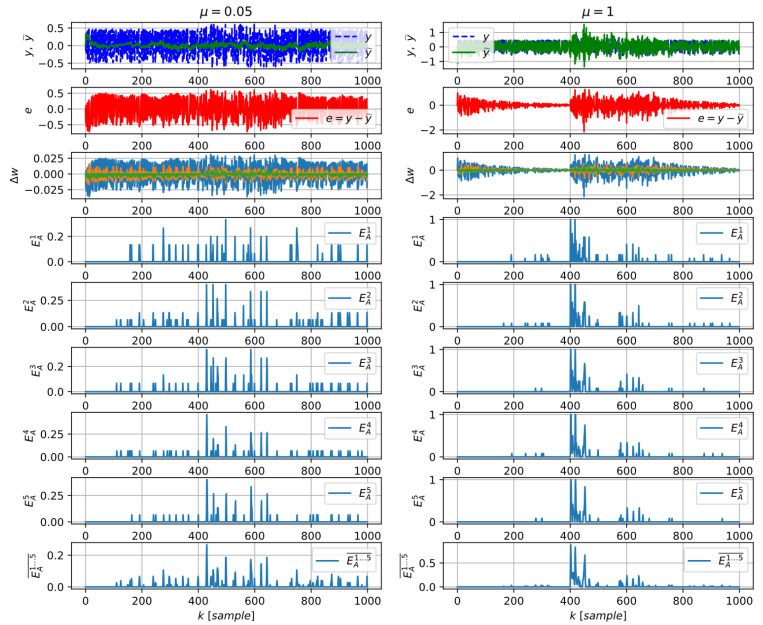
The performance of the direct algorithm for estimation of learning entropy of various orders (23) for not pretrained adaptive predictor with a too low learning rate μ=0.05 (left graphs) and for reasonable learning rate μ=1 (right graphs); normally distributed noise is within k∈<400,750> (same as in Figure 1 and Figure 3).

**Figure 3:**
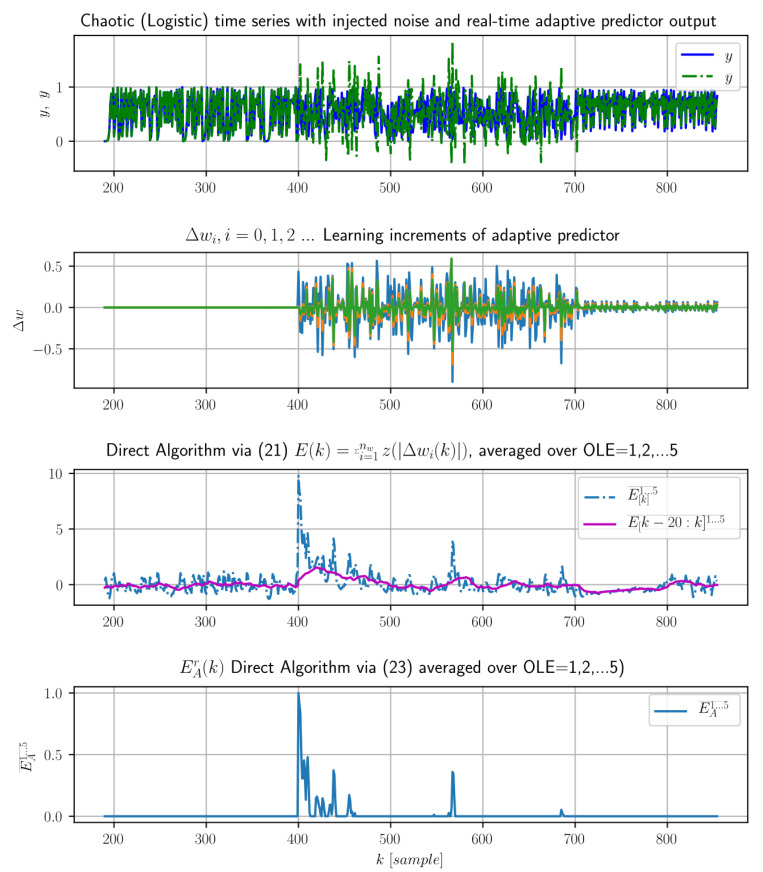
The limitation and challenges: The alternative LE estimation (21) displays capability to capture both unusually large learning effort as well as unusually small one, while the currently proposed algorithms of LE (18,19,23) are based on capturing unusually large learning effort and the novelty detection when the noise (400<k≤700) changes back to deterministic signal for k>700 is still a challenge. So far, we found the Direct Algorithm (23) (bottom axes) be practically comparable to the original LE estimation (19) (see Figure 1 with a similar type of data).

The performance of the direct algorithm (23) is demonstrated in Figure 2 and Figure 3, and we found the performance fairly comparable to the previously introduced estimation of LE (19) as we compared it with the same learning system and the same (gradient descent) learning algorithm and similar data as in Figure 1.

## 5. Limitations and Further Challenges

At first, the main considerations for both the power and weakness of learning entropy are the choice of a proper learning system, the learning algorithm, and its setups. Thus, background knowledge and relevant skills with machine learning can crucially affect the performance of LE.

Second, the fundamental assumption for learning entropy is that a learning system that adapts its weights via Δw is already pretrained.

This is demonstrated both in Figure 1 and also in Figure 3 for k>700, where the adaptive predictor was pretrained on initial deterministic time series that suddenly changed to white noise for k∈〈400,700). Then, the noisy data samples at k>400 results in the immediate increase of learning effort, so the LE increases immediately after k=400. However; the complexity of pure white noise for k∈〈400,700) disables the learning system for retraining, so the weight increments do not converge at all yet the LE decreases because the learning pattern is usually found within k∈〈400,700). Thus, when the data changes back to deterministic ones for k>700 (Figure 3), the LE fails to detect this novelty (bottom axis Figure 3) because the adaptive predictor was not retrained due to extreme complexity of the preceding signal (noise) and thus the new data for k>700 do not induce the increased learning effort.

The previously demonstrated limitation of LE (18,19,23) is based on a theoretical example, and so far, we have not encountered this issue in our research with deterministic systems or with real-world data. However, this theoretical case certainly demonstrates the challenge for further enhancement of algorithms for estimation of LE.

In future research, the intermediate alternative of the direct algorithm (21) shall be investigated as it can capture both suddenly increased learning effort and, with some latency due to floating averaging, also the immediate decrease of learning effort (Figure 3, middle axes, *k* > 700). Also, it is important to study LE with learning systems with more powerful learning criteria such as the ones employing generalized entropies (square-error based learning criteria have been investigated so far).

## 6. Conclusions

The main finding is that the learning effort of a pretrained learning model is quantifiable as a new (machine) learning-oriented information measure for each individually observed sample of data of otherwise complex dynamic systems while the model accuracy is not a necessary requirement for novelty detection. The method and the obtained results present LE as a cognitive concept of real-time novelty detection, where new information in data can be quantified via unusual learning effort, while, in principle, the error of the learning systems itself is not substantial. Being relieved from the assumption that model errors and novelty in data must be correlated, LE has the potential to detect novelty in complex behavior even with the use of imprecise learning systems. Thus, LE establishes a novel concept for research of new cognitive information measures with prospects to adaptive signal processing and cognitive computational intelligence methods with the very essence of learning systems.

## Figures and Tables

**Table 1:** Order of learning entropy (OLE) is determined by the difference in the order of weight increments in (12)–(14).

$E^{r}$ $r^{th}\mathrm{OLE}$	Detection Function Modifications for Varying Orders of LE
$E^{0}$	$L(\alpha )=\sum_{\forall \omega }f\left(\left|\left|\Delta \omega \right|-\bar{\left|\Delta w\right|}\right|>\alpha \cdot \sigma_{\Delta \omega }\right)$
$E^{1}$	$L(\alpha )=\sum_{\forall \omega }f\left(\left|\left|\Delta \omega \right|-\bar{\left|\Delta w\right|}\right|>\alpha \cdot \sigma_{\Delta \omega }\right)$ Δw(k)=w(k)−w(k−1)
$E^{2}$	$L(\alpha )=\sum_{\forall \omega }f(|\left|\Delta^{2}\omega \right|-\bar{\left|\Delta^{2}w\right|}|>\alpha \cdot \sigma_{\Delta \omega })$ $\Delta^{2}w(k)=\Delta w(k)-\Delta w(k-1)$
$E^{r}$	$L(\alpha )=\sum_{\forall \omega }f\left(|\left|\Delta^{r}\omega \right|-\bar{\left|\Delta^{r}w\right|}|>\alpha \cdot \sigma_{\Delta \omega }\right)$ $\Delta^{r}w(k)=\Delta^{r-1}w(k)-\Delta^{r-1}w(k-1)$

## References

[1] Shannon C.E. (1948). A mathematical theory of communication. Bell Syst. Tech. J..

[2] Markou M., Singh S. (2003). Novelty detection: A review—Part 1: Statistical approaches. Signal Process..

[3] Markou M., Singh S. (2003). Novelty detection: A review—Part 2: Neural network based approaches. Signal Process..

[4] Pincus S.M. (1991). Approximate entropy as a measure of system complexity. Proc. Natl. Acad. Sci. USA.

[5] Richman J.S., Moorman J.R. (2000). Physiological time-series analysis using approximate entropy and sample entropy. Am. J. Physiol. Heart Circ. Physiol..

[6] Kinsner W. (2007). Towards cognitive machines: Multiscale measures and analysis. Int. J. Cogn. Inf. Nat. Intel. (IJCINI).

[7] Kinsner W. (2007). A Unified Approach To Fractal Dimensions. Int. J. Cogn. Inf. Nat. Intel. (IJCINI).

[8] Kinsner W. (2007). Is Entropy Suitable to Characterize Data and Signals for Cognitive Informatics?. Int. J. Cognit. Inform. Nat. Int. (IJCINI).

[9] Zurek S., Guzik P., Pawlak S., Kosmider M., Piskorski J. (2012). On the relation between correlation dimension, approximate entropy and sample entropy parameters, and a fast algorithm for their calculation. Phys. A Stat. Mech. Appl..

[10] Schroeder M.R. (1991). Fractals, Chaos, Power Laws: Minutes from an Infinite Paradise.

[11] Costa M., Goldberger A.L., Peng C.-K. (2002). Multiscale Entropy Analysis of Complex Physiologic Time Series. Phys. Rev. Lett..

[12] Costa M., Goldberger A.L., Peng C.-K. (2005). Multiscale entropy analysis of biological signals. Phys. Rev. E.

[13] Wu S.-D., Wu C.-W., Lin S.-G., Wang C.-C., Lee K.-Y. (2013). Time series analysis using composite multiscale entropy. Entropy.

[14] Faes L., Nollo G., Porta A. (2013). Compensated transfer entropy as a tool for reliably estimating information transfer in physiological time series. Entropy.

[15] Yin L., Zhou L. (2013). Function based fault detection for uncertain multivariate nonlinear non-gaussian stochastic systems using entropy optimization principle. Entropy.

[16] Vorburger P., Bernstein A. Entropy-based Concept Shift Detection. Proceedings of the Sixth International Conference on Data Mining (ICDM’06).

[17] Amigó J., Balogh S., Hernández S. (2018). A Brief Review of Generalized Entropies. Entropy.

[18] Bereziński P., Jasiul B., Szpyrka M. (2015). An Entropy-Based Network Anomaly Detection Method. Entropy.

[19] Gajowniczek K., Orłowski A., Ząbkowski T. (2018). Simulation Study on the Application of the Generalized Entropy Concept in Artificial Neural Networks. Entropy.

[20] Ghanbari M., Kinsner W. Extracting Features from Both the Input and the Output of a Convolutional Neural Network to Detect Distributed Denial of Service Attacks. Proceedings of the 2018 IEEE 17th International Conference on Cognitive Informatics & Cognitive Computing (ICCI*CC).

[21] Willsky A.S. (1976). A survey of design methods for failure detection in dynamic systems. Automatica.

[22] Gertler J.J. (1988). Survey of model-based failure detection and isolation in complex plants. IEEE Control Syst. Mag..

[23] Isermann R. (1984). Process fault detection based on modeling and estimation methods—A survey. Automatica.

[24] Frank P.M. (1990). Fault diagnosis in dynamic systems using analytical and knowledge-based redundancy: A survey and some new results. Automatica.

[25] Widmer G., Kubat M. (1996). Learning in the presence of concept drift and hidden contexts. Mach. Learn..

[26] Polycarpou M.M., Helmicki A.J. (1995). Automated fault detection and accommodation: A learning systems approach. IEEE Trans. Syst. Man Cybern..

[27] Demetriou M.A., Polycarpou M.M. (1998). Incipient fault diagnosis of dynamical systems using online approximators. IEEE Trans. Autom. Control.

[28] Trunov A.B., Polycarpou M.M. (2000). Automated fault diagnosis in nonlinear multivariable systems using a learning methodology. IEEE Trans. Neural Netw..

[29] Alippi C., Roveri M. (2008). Just-in-Time Adaptive Classifiers—Part I: Detecting Nonstationary Changes. IEEE Trans. Neural Netw..

[30] Alippi C., Roveri M. (2008). Just-in-Time Adaptive Classifiers—Part II: Designing the Classifier. IEEE Trans. Neural Netw..

[31] Alippi C., Boracchi G., Roveri M. (2013). Just-In-Time Classifiers for Recurrent Concepts. IEEE Trans. Neural Netw. Learn. Syst..

[32] Alippi C., Ntalampiras S., Roveri M. (2013). A Cognitive Fault Diagnosis System for Distributed Sensor Networks. IEEE Trans. Neural Netw. Learn. Syst..

[33] Grossberg S. (2013). Adaptive Resonance Theory: How a Brain Learns to Consciously Attend, Learn, and Recognize a Changing World. Neural Netw..

[34] Bukovsky I. (2013). Learning Entropy: Multiscale Measure for Incremental Learning. Entropy.

[35] Bukovsky I., Oswald C., Cejnek M., Benes P.M. Learning entropy for novelty detection a cognitive approach for adaptive filters. Proceedings of the Sensor Signal Processing for Defence (SSPD).

[36] Bukovsky I., Homma N., Cejnek M., Ichiji K. Study of Learning Entropy for Novelty Detection in lung tumor motion prediction for target tracking radiation therapy. Proceedings of the 2014 International Joint Conference on Neural Networks (IJCNN).

[37] Bukovsky I., Cejnek M., Vrba J., Homma N. Study of Learning Entropy for Onset Detection of Epileptic Seizures in EEG Time Series. Proceedings of the 2016 International Joint Conference on Neural Networks (IJCNN).

[38] Bukovsky I., Oswald C., Silhavy R., Senkerik R., Oplatkova Z., Prokopova Z., Silhavy P. (2015). Case Study of Learning Entropy for Adaptive Novelty Detection in Solid-fuel Combustion Control. Intelligent Systems in Cybernetics and Automation Theory (CSOC 2015).

[39] Cejnek M., Bukovsky I. (2018). Concept drift robust adaptive novelty detection for data streams. Neurocomputing.

[40] Brissaud J.-B. (2005). The meanings of entropy. Entropy.

[41] Bukovsky I. Modeling of Complex Dynamic Systems by Nonconventional Artificial Neural Architectures and Adaptive Approach to Evaluation of Chaotic Time Series. PhD thesis (in English).

[42] Bukovsky I., Bila J., Wang Y., Zhang D., Kinsner W. (2010). Adaptive Evaluation of Complex Dynamical Systems Using Low-Dimensional Neural Architectures. Advances in Cognitive Informatics and Cognitive Computing. Studies in Computational Intelligence.

[43] Bukovsky I., Kinsner W., Bila J. Multiscale analysis approach for novelty detection in adaptation plot. Proceedings of the Sensor Signal Processing for Defence (SSPD 2012).

